# A Novel Homodimer Peptide–Drug Conjugate Improves the Efficacy of HER2-Positive Breast Cancer Therapy

**DOI:** 10.3390/ijms24054590

**Published:** 2023-02-27

**Authors:** Shurong Liu, Ye Tian, Sujun Jiang, Zihua Wang

**Affiliations:** Fujian Provincial Key Laboratory of Brain Aging and Neurodegenerative Diseases, School of Basic Medical Sciences, Fujian Medical University, Fuzhou 350122, China

**Keywords:** HER-2, breast cancer, peptide–drug conjugate, doxorubicin, targeted delivery

## Abstract

Tumor-targeting peptide–drug conjugates (PDCs) have become a focus of research in recent years. However, due to the instability of peptides and their short in vivo effective half-life, they have limited clinical application. Herein, we propose a new DOX PDC based on a homodimer HER-2-targeting peptide and acid-sensitive hydrazone bond, which could enhance the anti-tumor effect of DOX and reduce systemic toxicities. The PDC could accurately deliver DOX into HER2-positive SKBR-3 cells, with it showing 2.9 times higher cellular uptake than free DOX and enhanced cytotoxicity with respect to IC_50_ of 140 nM (vs. 410 nM for free DOX). In vitro assays showed that the PDC had high cellular internalization efficiency and cytotoxicity. In vivo anti-tumor experiments indicated that the PDC could significantly inhibit the growth of HER2-positive breast cancer xenografts in mice and reduce the side effects of DOX. In summary, we constructed a novel PDC molecule targeting HER2-positive tumors, which may overcome some deficiencies of DOX in breast cancer therapy.

## 1. Introduction

Breast cancer is one of the most common malignancies observed in women worldwide, with a large number of new cases and deaths every year. Human epidermal growth factor receptor 2 (HER2)-positive breast cancer exhibits stronger invasion and metastasis, a poorer prognosis, and a lower overall survival rate [1]. For HER2-positive breast cancer patients, anti-HER2 targeted drug intervention can reduce tumor aggressiveness and improve patient prognosis [2]. A variety of new therapeutics targeting HER2, including bispecific antibodies, antibody-drug conjugations (ADC), and immunotherapies, have also entered the clinical testing stage and shown great clinical application potential, which will provide rich options for the treatment of HER2-positive breast cancer [3]. Chemotherapy is still one of the main ways to treat breast cancer and small-molecule chemotherapy drugs are widely used in clinical cancer treatment. However, due to the lack of tumor-targeting therapy and the low bioavailability of chemotherapy, systemic toxicity and drug resistance often occur, which seriously affect their clinical applications. Therefore, methods for correctly delivering sufficient chemotherapeutic drugs to tumor tissue while reducing toxic damage to healthy tissue have become a focus of research with respect to drug delivery in breast cancer therapy. DOX, an anthracycline, is a commonly used drug in treating various types of cancer, such as breast cancer. Nevertheless, its nonspecific tissue distribution can lead to adverse effects, such as cardiotoxicity, myelosuppression, and nausea, which restrict its clinical application [4]. To enhance the anti-tumor effect of DOX and reduce its systemic toxicity, a range of strategies are employed [5,6]. Targeted ligands are often employed as a strategy to deliver chemotherapeutic agents to the cancer site, thus reducing the amount of enrichment in normal cells and tissues [7,8]. To date, antibodies, peptides, and other targeted ligands can be covalently attached to chemotherapeutic agents or drug delivery systems, such as ADCs, liposomes, and micelles, to enable the precise administration of drugs [9]. Nevertheless, the large molecular size of antibodies leads to ADCs with limited permeability in tumors, thus significantly reducing their therapeutic efficacy. Peptides can be easily prepared on a large scale using chemical synthesis, and they can be easily modified. Compared with ADCs, a peptide–drug conjugate (PDC) has the following advantages: higher drug loading, enhanced penetration ability in solid tumors, multifunctional modifications can be easily carried out using chemical methods, a wider selection of carrier drugs, and lower production costs [10,11,12,13]. Recently, a large number of PDCs have been introduced as potential diagnostics and anti-cancer drugs in the clinic [14,15]: this includes ^177^Lu-DOTA-octreotate, which was approved by the US Food and Drug Administration (FDA) in 2018 [16]. Therefore, targeted peptide-based drug delivery methods will provide a new impetus for cancer therapy in the future [17,18]. With the help of peptides, the drug can easily cross the cell membrane and reach the target site, reducing the toxic side effects of the non-specific uptake of tissues [19]. By combining the computational-aided OBOC peptide library design with in situ single-bead sequencing microarray methods, Wang and their team were able to successfully screen two peptide sequences, H6 and H10, which specifically bind to the HER2 receptor [20]. A preliminary clinical study in patients also showed that H10 peptide probe-based SPECT imaging could clearly reflect the HER2 status in guiding personalized therapeutic strategies for HER2-positive breast cancer patients [21].

Although PDCs have many advantages, their application as a therapeutic is still restricted because of their quick metabolism clearance and poor circulation stability in vivo [22]. Peptide multimerization has been demonstrated to be an efficient way of enhancing receptor binding affinity, mainly due to the “multivalent effect” or “apparent synergistic affinity” [23,24]. In addition, peptide trimers or tetramers have been reported to have significantly increased binding affinity to targets compared to their monomers and dimers [25,26]. Therefore, the optimized construction of a homodimer HER2 peptide, which is made up of two identical binding ligands connected via a linker, could synergically assist in a cooperative manner in the improvement of binding avidity with respect to addressing some drawbacks in PDCs [25,27,28]. In this study, we developed a PEGylated reversed H10 homodimer peptide as a novel HER2 peptide–drug conjugate (PDC) to improve tumor-targeting capabilities and anti-cancer activities.

## 2. Results and Discussion

### 2.1. Synthesis and Characterization of the PDC

**Figure 1:**
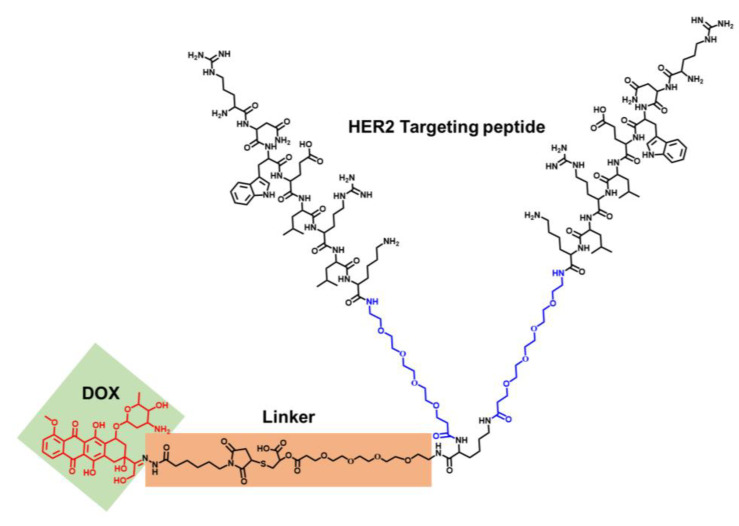
The structures of the HER2-targeting peptide–DOX conjugate (PDC).

To better realize chemotherapeutics’ targeted delivery and anti-tumor treatments, a homodimer peptide was designed with two PEG4 spacers between the two reversed monomeric H10 peptide motifs and one PEG4 linker in the cysteine α-amino group. To avoid potential steric hindrances, a PEG4 spacer was designed and subsequently reacted with a cysteine at the carboxyl terminus and a homodimer peptide (RNWELRLK-PEG4)2, forming HP. Then, the elongated thiol-modified homodimer peptide was conjugated with DOXO-EMCH in a Michael addition, forming a novel peptide-DOX PDC (Figure 1 and Figure S1).

**Figure 2:**
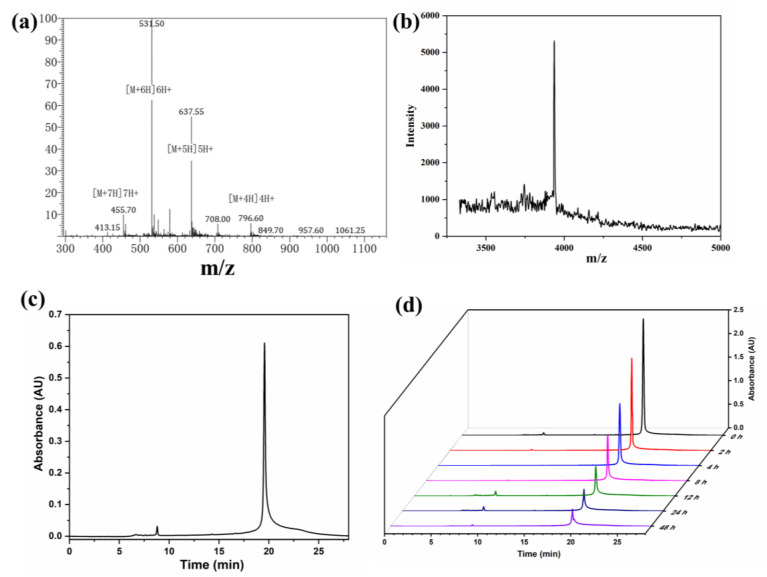
(**a**) Representative electrospray ionization (ESI) mass spectra of HP peptides. (**b**) MALDI-TOF mass spectrometry of the PDC. (**c**) Analysis and purification of the PDC by HPLC. (**d**) The stability of the PDC evaluated by HPLC.

HP (calcd Mw: 3183.7) was successfully synthesized by using solid-phase peptide synthesis (SPPS), and the peptides were cleaved and purified by HPLC (Figure S2) afterward and verified by using electrospray ionization mass spectrometry examinations (Figure 2a). As shown in Figure 2b,c, the PDC was synthesized and characterized by HPLC (wavelength at 490 nm) and mass spectrometry (calcd Mw: 3935.5). The mass charge ratio (m/z) obtained by the experiment is consistent with the theoretical m/z. The mass spectrometry results revealed that the products were successfully achieved. Polyethylene glycol (PEG) has been widely used to modify small molecules to slow renal clearance and to increase the half-life [29]. It is essential that the PDC be maintained in a steady circulation to prevent pre-release of cytotoxic payloads, resulting in systemic toxicity. This study demonstrates that the covalent attachment of PEG chains to peptides can generate new pharmacokinetic characteristics and increase the hydrophilicity of the peptides, thus decreasing uptake in non-target organs. To assess plasma stability, the PDC was incubated with human serum at 37 °C from 0 to 48 h, and aliquots were taken at different time points and analyzed by using HPLC. As shown in Figure 2c, the HPLC peak for the conjugate slowly decreased over time with a half-life of around 24 h in media which ensures the relative stability of the conjugate during blood circulation. The percentage of intact PDC decreased with an increase in incubation time, and about 15% of the intact conjugate remained at 48 h.

### 2.2. In Vitro Drug Release and Cytotoxicity

**Figure 3:**
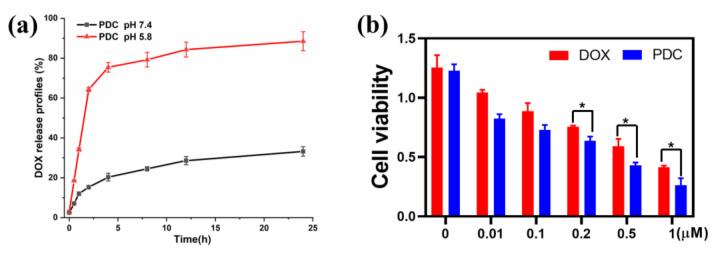
(**a**) In vitro release profiles of the PDC at pH 5.8 and 7.4. (**b**) Cell viability of SKBR-3 cells incubated with the PDC and free DOX for 48 h. Errors were determined with one-way analysis of variance (ANOVA). * *p* < 0.05 indicate statistical significance.

Based on the specific acidic tumor microenvironment (pH5.4–7.0), an acid-responsive linker was designed to undergo acid hydrolysis after cellular uptake, releasing the drug into endosomes or lysosomes to achieve site-specific delivery [30]. It is theoretically possible that a HPD with an acid-sensitive hydrazone bond can display a regulated release behavior when the external pH value is altered. As observed from the drug release profile in Figure 3a, the PDC remains structurally stable in PBS at a pH of 7.4, as only about 35% of DOX is released within 24 h. In contrast, when the pH of PBS is reduced to 5.8, there is an explosive release of DOX in acidic media due to the pH-sensitive hydrolysis of the hydrazine bond between DOX and the EMCH linker. The DOX release from the PDC was rapid, rising from around 20% to 78% within 8 h and nearing 90% within 24 h, indicating that the PDC has acid sensitivity and a controlled release, thus reducing harm to healthy cells. Figure 3b illustrates that the cytotoxicity of the PDC and free DOX on SKBR-3 cells augmented with prolonged incubation time. When the equivalent concentration was 1 μM, the viability of SKBR-3 cells in the PDC group was 26.3%. The IC_50_ values of the PDC for SKBR-3 cells calculated from the dose response log curves were 143 nM. The values were significantly lower than those of free DOX (410.5 nM for SKBR-3 cells). The IC_50_ of the PDC was three times less than that of free DOX. At the same time, it was observed that the inhibitory effect of the PDC on SKBR-3 cells was significantly stronger than DOX at all drug concentrations. To determine if our novel conjugate could enhance the effect of DOX through PEGylation and HER2-receptor-mediated internalization, we tested the cytotoxic effect of the PDC on MCF-7 and SKBR-3 breast cancer cells. We found that the PDC was significantly more cytotoxic in the HER2 high-expression cell lines (Figure S4). Obviously, the linked HER2 peptide greatly enhanced the tumor-cell-killing ability of doxorubicin.

### 2.3. In Vitro Intracellular Uptake

**Figure 4:**
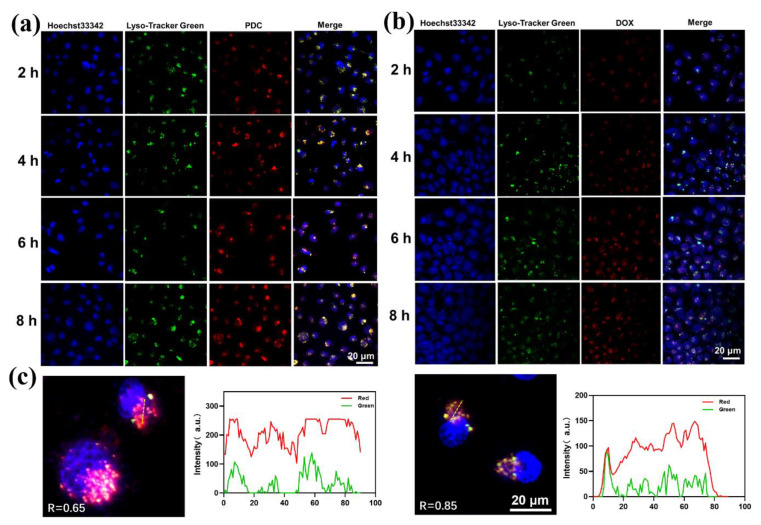
(**a**) CLSM imaging of the SKBR-3 cells’ uptake of the PDC. (**b**) CLSM imaging of the SKBR-3 cells’ uptake of free DOX. Blue, green, and red channels stand for the fluorescence of Hoechst33342, LysoTracker, and DOX, respectively. The scale bar is 20 µm. (**c**) Representative fluorescence images showing the colocalization of the PDC or DOX with the LysoTracker for 8 h. Red: DOX. Green: LysoTracker. Blue: Hoechst 33342. Fluorescence intensity analyses of pixels along the dotted line were measured by ImageJ.

To assess the uptake efficiency of the PDC in tumor cells, human breast cancer cell lines SKBR-3 (high expression of HER2) were used as positive cells, and the MCF-7 cell line was used as a negative control. The results from the confocal laser scanning microscope (CLSM) revealed a strong red fluorescence signal of the PDC on SKBR-3 after 2 h, suggesting that the endocytosis process which was mediated by the ligand was successful in attaching the PDC. Along with the incubation time, DOX escaped from the lysosomes, and brighter red fluorescence was detected in the nuclei after 8 h of incubation (Figure 4a). It has been observed that peptide conjugates in the acidic environment of endosomes/lysosomes undergo hydrolysis at the acid-response hydrazone linker, thereby releasing DOX into cells, which is in line with prior reports. In the free DOX group, most DOXs were still in the cytoplasm, revealing lower transport efficiency (Figure 4b). These results indicated that the PDC delivered substantially more DOX to SKBR-3 cells that are rich in HER2 receptors than free DOX itself. The lysosomal escape ability was evaluated by the localization of the PDC and lysosomes in SKBR-3 cells (Figure 4c), evidenced by the separation of red (DOX) and green (LysoTracker) fluorescence. It showed that the overlay signal of DOX with the endo/LysoTracker remained after colocalization at 8 h (R = 0.85), while the PDC showed little colocalization with the lysosomes (R = 0.65). These results demonstrated that the hydrazone linker was able to be cleaved from the PDC in endo/lysosomes to facilitate intracellular drug delivery. As shown from Figure S3, SKBR-3 cells have a higher uptake rate of the PDC than negative MCF-7 cells due to the difference in HER2 protein expression. Collectively, the above results suggest that the PDC that we designed was more effective in transporting DOX molecules into SKBR-3 cells, thereby increasing intracellular drug concentrations and enhancing anti-tumor effects.

### 2.4. In Vivo Anti-Tumor Studies

**Figure 5:**
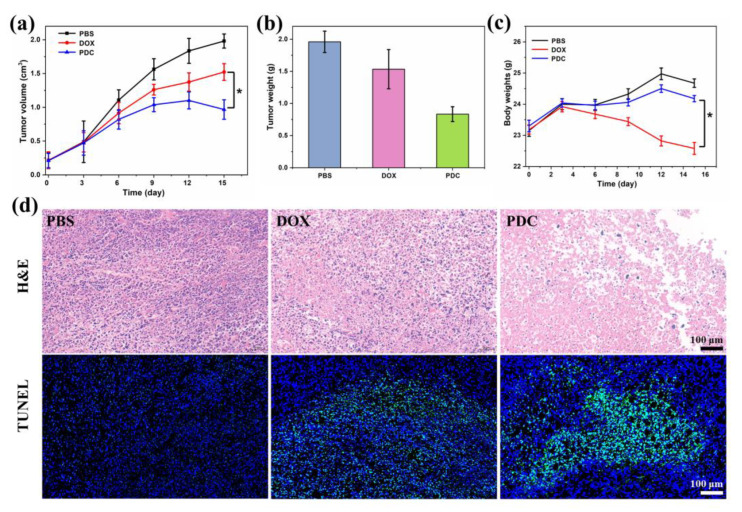
In vivo anti-tumor effect of the PDC. (**a**) Tumor growth curves after the treatment of saline, free DOX, and the PDC. (**b**) Tumor weight after intravenous administration. (**c**) Body weight change curves. (**d**) Analysis of the tumor slices with H&E and TUNEL staining in different therapeutics. Errors were determined with one-way analysis of variance (ANOVA). * *p* < 0.05 indicate statistical significance.

In vivo anti-tumor studies were evaluated by using SKBR-3 xenografted (BALB/c nude) mice treated with the PDC, free DOX, or saline. Figure 5a demonstrates that the PDC had a much more powerful anti-tumor effect than free DOX, reducing tumor growth by 51.1 ± 3.1% on day 14 post treatment, while free DOX only achieved a 23.13 ± 2.4% reduction. Additionally, the PDC had a significantly higher tumor weight inhibition of 57.5  ±  3.4% compared to free DOX (Figure 5b). Figure 5c demonstrated that there was no noteworthy distinction in body weight between the PDC group and PBS group, whereas the weight of the free DOX group significantly decreased, likely due to the systemic toxicity caused by the free DOX. The results showed that the PDC had good biocompatibility and safety in vivo. Additionally, histological examination of the tumors was performed. After PDC treatment, H&E staining of the tumor tissues showed a large number of necrotic areas and tumor cell apoptosis. A TUNEL assay indicated a greater number of apoptotic cells after treatments with the PDC group compared to the free DOX group and reduced the offtarget effects (Figure 5d). These data illustrated that the PDC enhanced drug delivery with respect to tumor sites and exerted a powerful anti-tumor effect against HER2-positive tumors in vivo.

**Figure 6:**
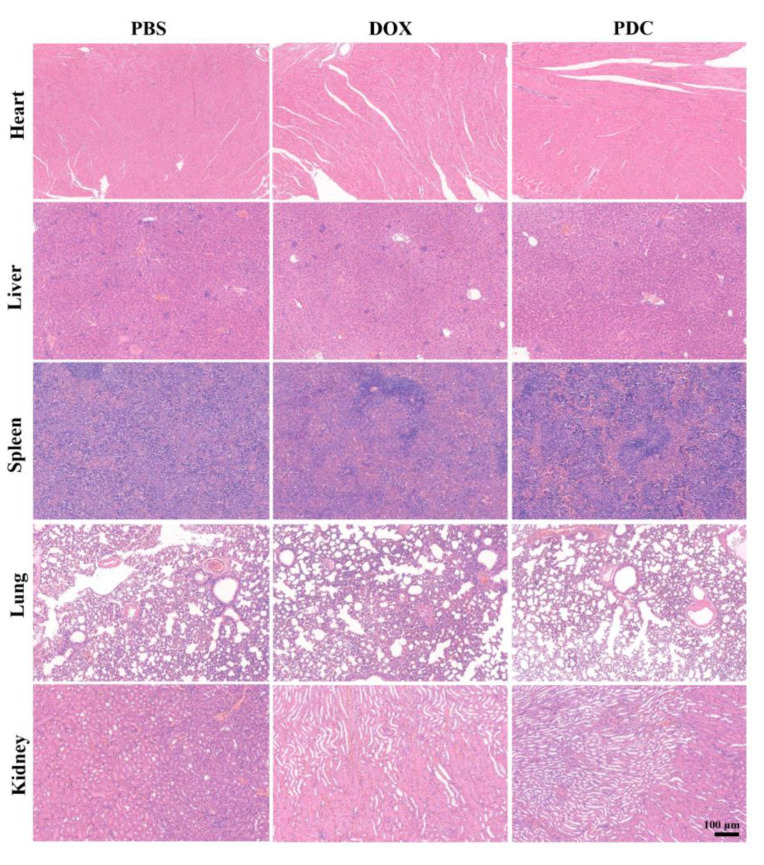
H&E staining of the major organs harvested from the mice of different treatment groups. Scale bar, 100 μm.

Good biocompatibility and the acceptable biosafety of a drug are essential for its biological application. Figure 6 revealed no morphological aberrations in organ tissues between the PBS and PDC treatment groups, suggesting no toxicity to these tissues. In contrast, the free DOX group exhibited clear cardiotoxicities, including extensive irregular arrangement and cell shrinkage. The PDC had higher in vivo anti-tumor efficacy than DOX without apparent toxicities. In all of the observations, the PDC showed significant tumor growth inhibition in vivo with no systemic toxicity and minimal cardiotoxicity. In conclusion, the design of peptide-based conjugates has obvious advantages in precise and targeted drug delivery and a reduction in systemic toxicity with respect to DOX.

## 3. Materials and Methods

### 3.1. Reagents and Antibodies

A CCK8 kit, doxorubicin hydrochloride, and the (6-maleimidocaproyl) hydrazone of DOX (DOXO-EMCH) were purchased from Med Chem Express LLC Co., Ltd. (Shanghai, China). DMEM/high glucose medium and trypsin were procured from GE Healthcare Life Sciences, while female BALB/c nude mice (8 weeks old) were obtained from Vital River Laboratory Animal Technology Co., Ltd. (Beijing, China). All other chemicals utilized in this study were purchased from J&K Scientific Ltd.

### 3.2. Preparation of HP-Peptide-Conjugated DOX

HP peptides were synthesized on Wang resin using standard Fmoc solid-phase peptide synthesis procedures. Following TFA-based cleavage from the resin, the crude peptides were purified and characterized by high-performance liquid chromatography (HPLC) and a matrix-assisted laser desorption ionization time-of-flight mass spectrum (MALDI-TOF-MS). A mixture of DOXO-EMCH (2.4 mM) in water/dimethylformamide (*v*:*v*, 1:1) and HP (1.2 mM) in PBS was prepared and kept at room temperature under stirring for 4 h [7]. Subsequently, the reaction mixture was dried under high vacuum and purified by RP-HPLC (method used: 10−70% with 0.01% TFA, flow rate = 1 mL/min, at 495 nm, absorbance maxima for aldoxorubicin).

### 3.3. In Vitro Drug Release and Stability Assay

To quantify the amount of DOX released from the PDC, 1 mg of the substance was dissolved in 2 mL of ultra-pure water and placed into a dialysis bag (MWCO 1000 Da). This bag was then transferred to a tube containing 10 mL of PBS solution with varying pH values (7.4 and 5.8). The tube was stirred at 37 °C in the dark and samples of the PBS solution were collected at different intervals. Subsequently, a new 10 mL of PBS was added to the tube after each sample. The samples were analyzed for released DOX concentration using a fluorescence spectrophotometer at 589 nm and the DOX concentration was calculated based on a standard curve. The PDC was incubated with human plasma (final peptide concentration of 50 μM) for 0, 2, 4, 8, 12, 24, and 48 h in order to assess the stability of the conjugate. Then, 50 mL of the solution was mixed with 150 mL of methanol to terminate enzymatic hydrolyses. After centrifugation at 13,000× *g* for 10 min, the supernatant was collected and the intact percentage of the PDC was detected by HPLC [19].

### 3.4. Cellular Uptake and Cytotoxicity Assay of the PDC

Cultures of SKBR-3 and MCF-7 cells were established in DMEM medium supplemented with 10% fetal bovine serum, 100 U/mL penicillin, and 100 μg/mL streptomycin at 37 °C and 5% CO_2_. Approximately 1 × 10^5^ cells/mL were seeded into culture dishes and incubated overnight. Subsequently, 0.5 mg/mL PDC was added and incubated for 2–8 h in conjunction with an equal amount of free DOX as a control. After washing three times with PBS, Hoechst 33342 and LysoTracker Green DND-26 were added to stain the nucleus and lysosome for 15 min, followed by three additional washes with PBS. Finally, confocal fluorescence images were obtained using a Zeiss 710 system. SKBR-3 cells were seeded in a 96-well culture plate at a density of 5000 cells per well and incubated for 24 h. Afterwards, cytotoxicity investigations were conducted by incubating the cells with the PDC and free DOX (in concentrations from 0 to 1 μM) for 48 h. Then, the CCK8 kit was used to detect cell survival.

### 3.5. In Vivo Anti-Tumor Study

In this study, a total of 12 female BALB/c mice (22–24 g) were used, and all animal experiments were conducted in compliance with the ethical guidelines approved by the ethics committee of Fujian Medical University. Approximately 2 × 10^6^ SKBR-3 cells were injected subcutaneously in the right hind limb of the nude mice. Upon the tumor reaching a volume of around 100 mm^3^, the mice were randomly allocated into three groups (n = 4) and administered through the tail vein with PBS, free DOX solution, and PDC solution (5 mg/kg equivalent DOX), respectively, five times every two days. Saline was used as a negative control. After each administration, the size and weight of the tumor were assessed. Subsequently, the mice were euthanized, and their tumor tissues and other major organs were dissected and preserved with 4% formaldehyde. Paraffin sections were then produced, and the fixed paraffin-embedded tissues were sliced into 5 μm sections for hematoxylin and eosin (H&E) and TUNEL (terminal deoxynucleotidyl transferase dUTP nick-end labeling) analyses.

## 4. Conclusions

From a clinical perspective, low DOX selectivity and cardiotoxicity during treatment limit its clinical use. Therefore, new research efforts are needed in order to improve the efficacy and safety of DOX [18]. To improve the inhibitory effect of DOX on tumors and to reduce its toxicity, various prodrugs and conjugations have been developed in recent years. Among them, peptide–drug conjugates have attracted substantial attention because of their superior biocompatibility, easy modification capabilities, degradability, and accessible preparation processes. To ensure that the drug is delivered to the intended site and can perform its designated role, peptide–drug conjugates are typically linked by cleavable structures [31]. Therefore, most peptide–drug conjugates are connected by cleavable structures. In summary, we successfully solved these problems and combined DOX with homodimer peptides to obtain novel PDC molecules, which not only improved the blood circulation half-life of DOX, but also achieved tumor-specific precision drug release. Furthermore, using a dimeric peptide for targeting offers potential advantages, including longer blood circulation times, improved specificity, and higher tumor penetration when compared with free DOX. The studies of cytotoxicity and endocytosis showed that HPD increased the cellular uptake of DOX and its inhibitory effect on SKBR-3 cells. PEGylation is a widely used strategy for improving the in vivo pharmacokinetics of PDCs. The incorporation of three PEG4 linkers in the HP peptide enhanced the pharmacokinetics properties of the peptide conjugate via the extension of its half-life. Taken together, our results indicate that using homobivalent ligands strategies could be a reliable method for enhancing binding affinity, which may result in improved the drug efficacy to inhibiting tumor growth. This presents a new targeted delivery strategy for obtaining various stable PDCs for anti-cancer therapy [32,33].

## Data Availability

Data available under request.

## Supplementary Materials

The following supporting information can be downloaded at: https://www.mdpi.com/article/10.3390/ijms24054590/s1.
