# Supplementary material for: A Novel Homodimer Peptide–Drug Conjugate Improves the Efficacy of HER2-Positive Breast Cancer Therapy
Source: Int J Mol Sci. 2023 Feb 27;24(5):4590. doi: 10.3390/ijms24054590 (PMC10003747; doi:10.3390/ijms24054590)
Supplement: Supplementary file 1 [file ijms-24-04590-s001.zip › ijms-2105923-supplementary.pdf]

A Novel Homodimer Peptide–Drug Conjugate Improves the Efficacy of HER2-Positive Breast Cancer Therapy

Shurong Liu†, Ye Tian†, Sujun Jiang and Zihua Wang*

Fujian Provincial Key Laboratory of Brain Aging and Neurodegenerative Diseases, School of Basic Medical Sciences, Fujian Medical University, Fuzhou 350122, China

* Correspondence: wangzh@fjmu.edu.cn

† These authors contributed equally to this work.

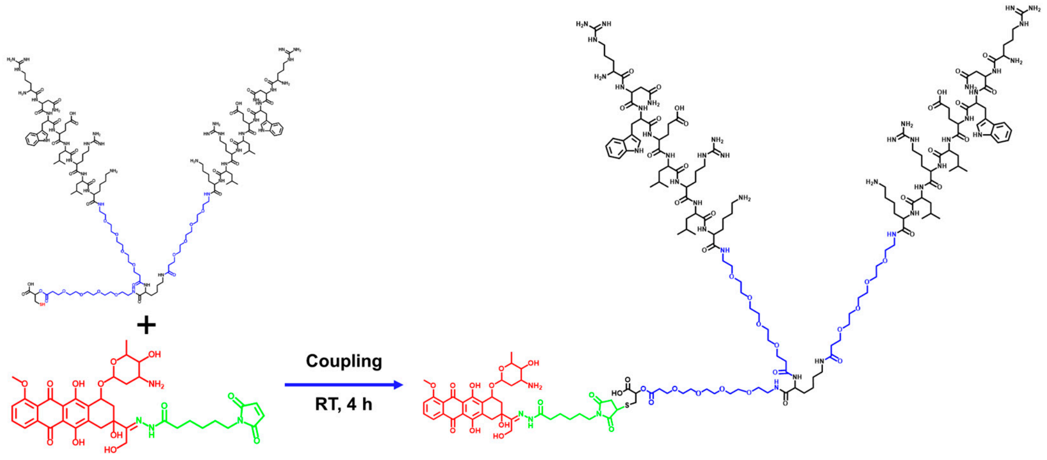

Figure S1. Scheme for doxorubicin–HP conjugate synthesis.

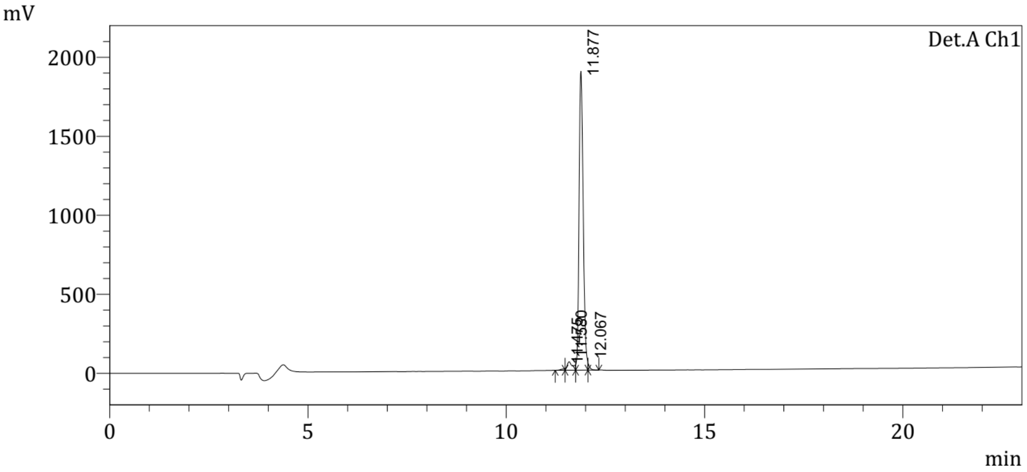

Figure S2. Analysis and purification of HP peptide using HPLC.

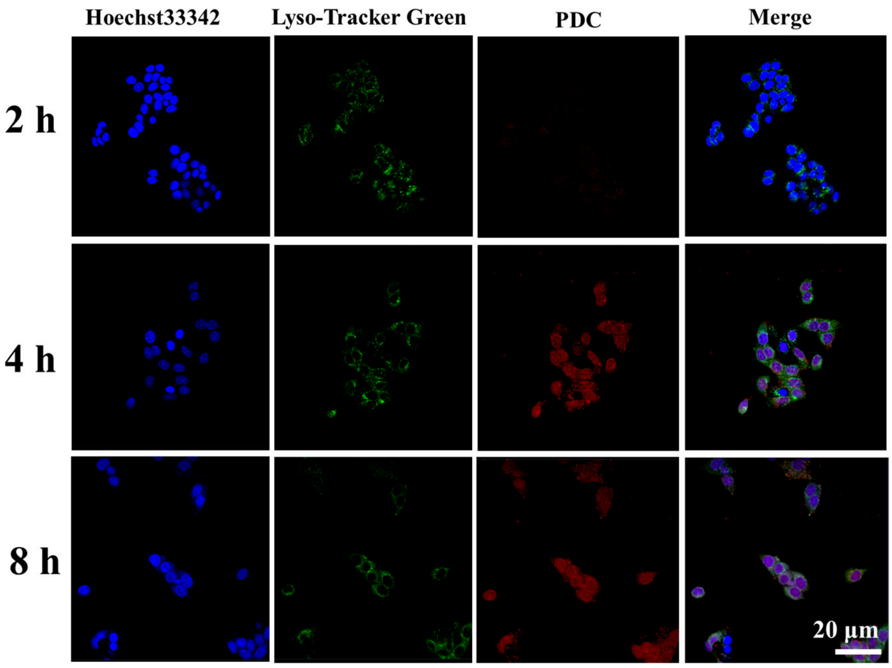

Figure S3. CLSM imaging of HER2 negative cell MCF-7 uptake of PDC. Scale bar is 20 μm.

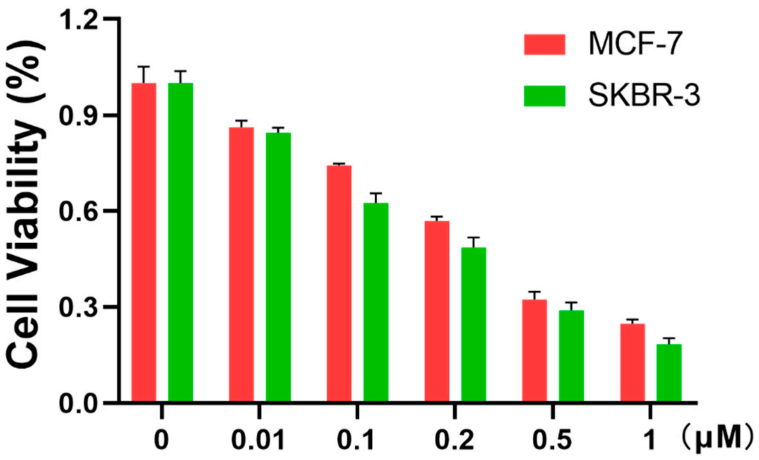

Figure S4. Cell viability of SKBR-3 and MCF-7 cells incubated with PDC for 48 h.
